# Recurrent Hodgkin’s Lymphoma Detected Using Abnormal NIPT in Pregnancy: A Case Report and Literature Review

**DOI:** 10.3390/diagnostics16101490

**Published:** 2026-05-14

**Authors:** Claudia Szlek, Puja Punukollu, Lindsey Grater, Debra Ware, Lawrence Devoe, Natalia Schlabritz-Lutsevich, Heidi David, William Toussaint, James Maher

**Affiliations:** 1 Department of Obstetrics and Gynecology, Medical College of Georgia, Augusta, GA 30912, USA; cszlek@augusta.edu (C.S.); mpunukollu@augusta.edu (P.P.); lgrater@augusta.edu (L.G.); hedavid@augusta.edu (H.D.); wtoussaint@augusta.edu (W.T.); 2 Section of Maternal Fetal Medicine, Department of Obstetrics and Gynecology, Medical College of Georgia, Augusta, GA 30912, USA; deware@augusta.edu (D.W.); ldevoe@augusta.edu (L.D.); 3 Advanced Fertility Institute, Texas Tech University Health Sciences Center, Permian Basin, Odessa, TX 79762, USA; natalia.schlabritz-lutsevich@ttuhsc.edu

**Keywords:** NIPT, prenatal diagnosis, maternal cancer, Hodgkin’s lymphoma, ICE chemotherapy, BEAM conditioning, autologous stem cell transplantation

## Abstract

**Background**: Non-invasive prenatal testing (NIPT) examines cell-free DNA (cfDNA) in maternal serum, which includes both maternal DNA and apoptotic placental DNA. The presence of multiple aneuploidies or widespread abnormal patterns of gains and losses across chromosomes in a structurally normal fetus has been linked to maternal cancer. **Case Presentation:** The patient was a 22-year-old G1P0 with a history of classical Hodgkin’s lymphoma in remission. Her NIPT collected at 14 weeks and 3 days was reported as a “no call”. A second NIPT at a different laboratory showed multiple chromosomal aneuploidies (trisomy 18, 21, and monosomy X) with normal fetal anatomy on ultrasound. The patient was asymptomatic and was referred to hematology–oncology specifically to address the concern that these NIPT results could be related to cancer recurrence. Imaging was deferred as she was already on an established surveillance protocol for her Hodgkin’s lymphoma. At 26 weeks of gestation, the patient presented with a cough and dyspnea. Chest x-ray raised concern for disease recurrence, and biopsy confirmed recurrent Hodgkin’s lymphoma. She received two cycles of ICE chemotherapy. Cesarean delivery at 34 weeks and 2 days was performed for non-reassuring fetal heart tones. She continued chemotherapy, followed by BEAM conditioning and autologous stem cell transplantation. Genetic testing of the neonate revealed a normal karyotype; the placenta karyotype yielded no interpretable results. **Discussion and Conclusions**: Certain patterns of abnormal NIPT results may be associated with maternal malignancy and warrant further investigation. The absence of standardized protocols for reporting such NIPT results can complicate timely interdisciplinary evaluation and treatment. However, diagnostic testing should be offered with a positive NIPT result, a no-call or test failure, and abnormal ultrasound results, even with a “low-risk” NIPT result.

## 1. Introduction

Molecular medicine has significantly transformed maternal aneuploidy screening during pregnancy. In 1997, Lo and colleagues published a landmark study documenting the presence of fetal cell-free DNA (cfDNA) in maternal circulation [1]. By 2011, this technology was available commercially as a screening method for fetal aneuploidy. The Non-Invasive Chromosomal Evaluation (NICE) Study, published in 2012, reported non-invasive prenatal testing (NIPT) results that were validated by a comparison with invasive prenatal testing results, demonstrating a significant improvement in the sensitivity and specificity of this cfDNA serum screening platform for fetal aneuploidy [2]. The following year, Osborn and colleagues reported a case of maternal metastatic neuroendocrine carcinoma in a woman who delivered a healthy baby despite cfDNA results indicating trisomy 13 and monosomy 18 [3]. This false positive suggested that in addition to fetal information, the test may also be able to provide insight into previously unrecognized maternal health conditions.

Several reports now discuss the possible causes for discrepancies when the fetal phenotype and cfDNA results differ [4]. Currently, three main next-generation sequencing (NGS) platforms are available for aneuploidy screening in pregnancy, with at least 13 companies offering screening options in the United States [5,6]. Each company uses proprietary bioinformatic algorithms to analyze the cfDNA data and report on the risk of fetal aneuploidy. When the test fails to yield informative “fetal results”, there is no consensus, standardization of terminology, or reporting protocols for these incidental or atypical test results [6]. As a result, healthcare providers ordering aneuploidy screening must be aware of the implications of the screening platform and reporting strategy for handling outcomes in the laboratories that they use. The heterogeneity in how NIPT results are reported can cause confusion, particularly because a “no call” result on NIPT can imply more than just a test failure, depending on the underlying cause. The provider must identify the reason for the atypical results to effectively counsel the patient. Test failure causes include sample issues, such as tube breakage or insufficient sample size; quality control issues with the assay, which prevent an informative results including interfering substances like heparin; and low fetal fraction, which has been linked to an increased risk of aneuploidy [7,8,9]. In addition to test failures, false positives may be due to maternal and placental contributions rather than fetal conditions. More complex findings, such as autosomal monosomy, multiple aneuploidies, globally chaotic data, and genome-wide sub chromosomal imbalances, have been associated with a higher risk of maternal cancer [6,10,11]. We report a case of Hodgkin’s lymphoma in remission for nearly a year before pregnancy, the recurrence of which was heralded by an abnormal first-trimester NIPT. At the time of her aneuploidy screening, she was asymptomatic. NIPT showed multiple autosomal aneuploidies, and the findings were linked to a possible maternal malignancy.

## 2. Case Presentation

**Figure 1:**
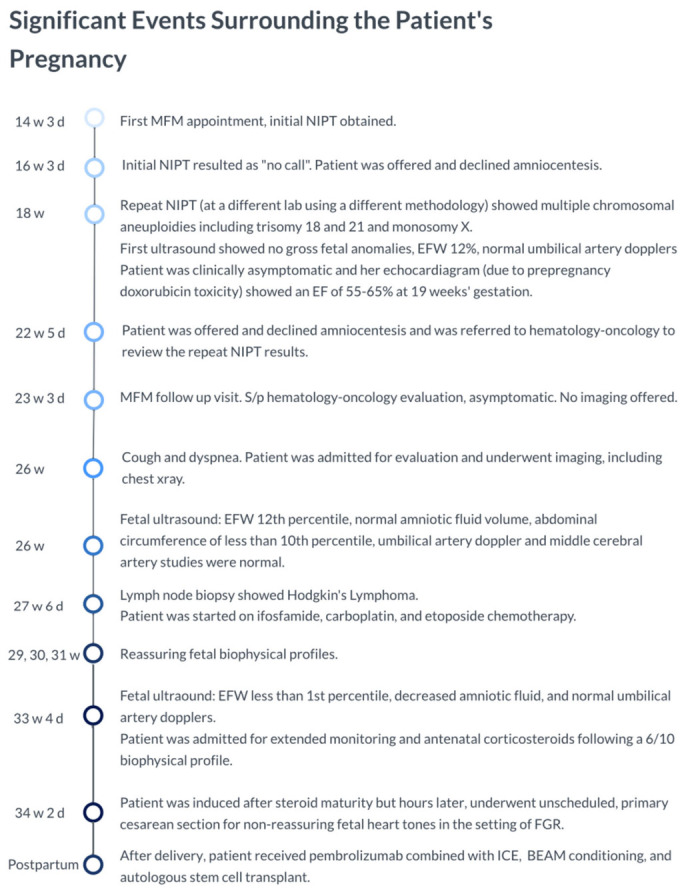
Significant events surrounding the patient’s pregnancy.

The patient was a 22-year-old G1P0 with a history of classical Hodgkin’s lymphoma (in remission; last treatment 8 months before conception) who presented to the Maternal Fetal Medicine (MFM) clinic at 14 weeks and 3 days’ gestation. The NIPT was obtained along with routine prenatal laboratory studies on this first visit (Figure 1).

**Figure 2:**
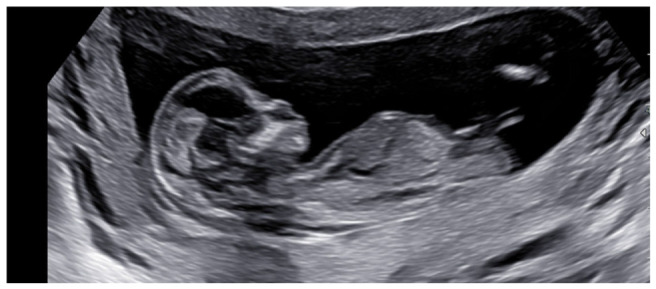
Normal first-trimester ultrasound.

The initial NIPT result at around 14 weeks’ gestation was a no-call result, and she was offered and declined amniocentesis. The anatomy ultrasound revealed a singleton pregnancy with normal amniotic fluid, typical fetal anatomy, no uterine fibroids, and an estimated fetal weight (EFW) at the 35th percentile (Figure 2).

A second NIPT, at around 18 weeks’ gestation, was performed through a different laboratory in the hopes that this would increase the informative results. The report showed multiple chromosomal aneuploidies, including trisomy 18, trisomy 21, and monosomy X. At 23 weeks and 3 days’ gestation, a follow-up ultrasound again showed no gross fetal anomalies. The EFW was now at the 12th percentile, with normal fluid volume and umbilical Doppler studies. The patient was counseled and offered amniocentesis again, which she declined once more. She was clinically asymptomatic during this visit, and an echocardiogram, conducted due to a pre-pregnancy history of doxorubicin cardiomyopathy, showed an ejection fraction of 55–65%. Referral to hematology–oncology was initiated after this visit because of concern that the multiple aneuploidies on the NIPT might indicate a recurrence of Hodgkin’s lymphoma. When she followed up with hematology–oncology, they confirmed that she remained asymptomatic from her prior cancer and declined to order early imaging as she was already on a surveillance protocol that was started with her prior cancer treatment. The next scheduled oncology check-up was not planned until after the anticipated delivery.

**Figure 3:**
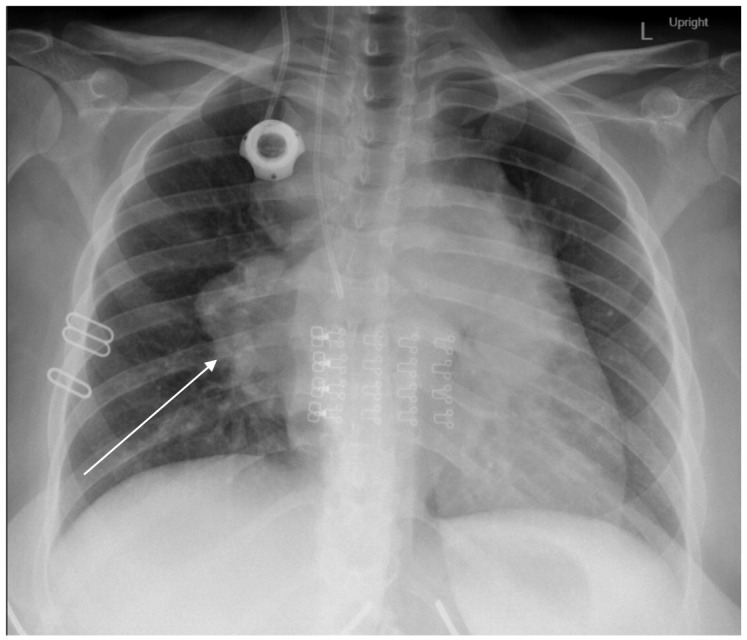
Patient’s chest x-ray at the time of diagnosis with an arrow showing bulky hilar adenopathy.

The patient presented for a follow-up MFM visit at 26 weeks with a cough and dyspnea. A chest x-ray showed bulky mediastinal and hilar lymphadenopathy (Figure 3). This imaging was suspicious for disease recurrence, and she was admitted to the hospital. A CT of the chest confirmed the presence of bulky mediastinal and bilateral hilar lymphadenopathy, as well as some infectious or inflammatory left ground-glass opacities, scattered pulmonary nodules, and hepatic steatosis. An ultrasound of the fetus at this time showed EFW at the 12th percentile, normal amniotic fluid volume, and an abdominal circumference of less than the 10th percentile. Umbilical artery doppler studies and middle cerebral artery doppler studies were normal for gestational age.

Interventional pulmonology performed a lymph node biopsy. The biopsy results were consistent with classical Hodgkin’s lymphoma. She was started on ifosfamide, carboplatin, and etoposide (ICE chemotherapy) and tolerated the three days of treatment. The hematology–oncology service was reluctant to treat her with pembrolizumab due to the potential risk of transplacental transfer to the fetus. Ultrasounds at 29, 30, and 31 weeks’ gestation revealed continued normal amniotic fluid and 10/10 biophysical profiles. The second cycle of ICE chemotherapy was administered 21 days later, and delivery was scheduled at 34 weeks’ gestation after a course of antenatal steroids had been administered. The estimated fetal weight (EFW) was less than the first percentile at 33 weeks and 4 days with decreased amniotic fluid volume and normal umbilical artery doppler studies. She was admitted for extended monitoring and the administration of antenatal corticosteroids. At 34 weeks and 2 days, she was induced after steroid maturity. However, just hours later, she underwent an unscheduled, primary cesarean section due to non-reassuring fetal heart tones in the setting of severe FGR. A female infant was delivered with Apgar scores of 2, 7, and 8, weighing 1050 g. After delivery, the patient received her third cycle of chemotherapy, which included pembrolizumab combined with ICE. The patient was also given Lupron to preserve fertility and subsequently underwent BEAM conditioning (carmustine, etoposide, cytarabine, and melphalan) and an autologous stem cell transplantation. Her infant spent 39 days in the NICU and was discharged in good condition. The patient has remained in clinical remission and is being closely monitored by the hematology–oncology and cardiology teams. Her infant is doing well and meeting developmental milestones.

## 3. Discussion

### 3.1. Screening

In medical screening tests, the sensitivity and specificity are characteristics of the test, and the positive predictive value (PPV) and negative predictive value (NPV) provide a direct assessment of the test’s performance in a population. The prevalence of the disease strongly influences the PPV and NPV [12].

Aneuploidy screening is predicated on an assessment of the a priori prevalence of aneuploidy and subsequent risk modifications based on specific features of the pregnancy. Aneuploidy screening has evolved from a history-based risk assessment to the incorporation of serum analytes that evaluate placental function, and then to the addition of ultrasound evaluation of the late first-trimester fetus in combination with the history and serum markers.

Next-generation sequencing (NGS) has revolutionized this screening process. Second-generation NGS technology involves the massively parallel sequencing of short DNA fragments (reads), combined with bioinformatics algorithms to analyze and interpret the sequence data and make predictions about the chromosome complement of the fetus. The allure of the NGS approach lies in the test’s higher sensitivity and specificity for common autosomal trisomy (CAT), such as 13, 18, and 21. Initially, NGS was recommended as a second-tier test for patients who were screened as having an elevated risk of aneuploidy with traditional screening. NIPT has now largely replaced serum analyte screening as the primary screening tool due to its higher sensitivity and specificity in both high- and low-risk patients.

### 3.2. Sequencing

Because sequencing costs are predicated on the number of base pairs analyzed, there is a trade-off between cost and sequencing depth. Sequence depth refers to the average number of reads aligning to a known reference at a specific location within the target transcript or genome. Sequencing is error-prone, requiring an increased number of reads to increase confidence in the called bases. Coverage indicates the proportion of the genome that was sequenced at least once, expressed as a percentage. For human genome sequencing, the standard coverage is 30×–50×, which strikes a balance between cost and data quality, while ensuring accurate variant detection.

With NIPT, for aneuploidy detection, we can make statistically valid predictions of chromosomal aneuploidy with significantly lower coverage and sequencing depth. Computational NIPT studies have indicated that the most critical reliability factor in NIPT analysis is sequencing depth. A higher sequencing depth represents a greater likelihood of comprehensive genome-wide interrogation and the detection of chromosome-spanning gains or losses. Currently, a sequencing depth of 10 Million reads per sample (RPS) is sufficiently reliable for clinical screening for risk of Down syndrome, Edwards syndrome, and Patau syndrome [13]. All the algorithms demonstrated a considerable increase in the number of false-negative trisomy findings as the number of reads fell below 5 Million RPS. The fetal fraction is particularly important at lower reads.

Only sequence reads that could be mapped to just one genome location in a reference human genome are retained after the data-filtering procedure (“unique reads”). The Illumina HiSeq platform and software used in the noninvasive prenatal diagnosis of trisomy 21, 18, and 13 generally produced ∼10 million 36-bp raw reads per sample, which is equivalent to just one-tenth of the total human genome, with only 20% (∼2 million) retained as unique reads. Deeper sequencing allows for finer resolution but at an additional cost. Genome-wide NIPT can expand the abnormalities that are detectable to include sex chromosome aneuploidy, rare autosomal trisomy (RATs), and sub chromosomal aberrations (MMS). There has been a push to extend the range of NIPT from CATs, such as trisomy 13, 18, and 21, to RATs and sub chromosomal CNVs as small as 3 Mb (MMS) in size by increasing the sequencing depth.

The maternal serum of a pregnant woman contains a mixture of maternal DNA, primarily derived from the breakdown of apoptotic maternal white blood cells, and a smaller contribution from placental trophoblast DNA (predominantly cytotrophoblasts). Placental DNA can be reliably detected in maternal serum as early as 6 weeks of gestation and is rapidly cleared from the maternal serum after delivery. The fetal fraction—the proportion of DNA from the placenta—increases with gestational age.

The prediction of the fetal chromosomal complement from the NIPT is predicated on a series of assumptions, which start with the assumption that the test is reliable in representing the DNA base pairs of the individual fragments. Some regions of the genome with extreme GC content (either very high or very low) may be more challenging to sequence, resulting in gaps in coverage. This can often be compensated for by increasing the overall coverage and depth of sequencing. For the test to be informative, there must be sufficient placental fragments to make statistically valid inferences, and the placental fragments must represent the true state of the fetus. A sample is considered aneuploid for a given region if it has a statistically significant deviation in the number of sequenced fragments (“depth”) relative to the disomic background samples and/or regions. An informative test result is reported as either high risk or low risk for aneuploidy.

### 3.3. Genome-Wide NIPT vs. Targeted

Several NIPT strategies are available, including whole genome sequencing, as well as the targeted enrichment of selective chromosomes. Laboratories utilize various methods for analyzing cfDNA, including massively parallel whole genome shotgun sequencing, chromosome-selective sequence analysis or the digital analysis of selected regions and single nucleotide polymorphism (SNP)-only-based analysis, microarray analysis, and rolling circle amplification (RCA). The semiconductor sequencing platform is a recent sequencing innovation associated with a lower sequencing cost and faster turnaround time.

Marton and colleagues recently highlighted the sensitivity, specificity, PPV, and NPV of several commercial laboratory NIPT tests in a systematic review. Zhen and colleagues also recently demonstrated the high sensitivity of genome-wide NIPT screening, which confirmed the findings in a meta-analysis of previous studies [14,15].

### 3.4. Positive Predictive Value

For clinical validity, the positive predictive value (PPV) is considered one of the most relevant test characteristics. The concept of PPV, however, is more nuanced for NIPT than for other tests. PPV depends on whether NIPT results are compared with the genotype of fetal or newborn cells, as opposed to the genotype in chorionic villi or placenta cells. NIPT is more sensitive in detecting trisomy than chorionic villus sampling (CVS), especially in cases of mosaicism, because NIPT is a “wash out” of the entire placenta whereas CVS provides a localized biopsy of a small region of the placenta. In a study of 10 cases where a rare autosomal trisomy (RAT) was detected using cfDNA and not confirmed in fetal or newborn cells, the RAT was, without exception, confirmed in placental biopsies at birth [16]. The gestation age at the time of the testing also affects the test results. NIPT gives access to first-trimester aneuploid pregnancies that are likely to fail. NIPT for all numerical chromosomal aberrations detects 10.8 times more aberrant cases than are expected to be born. For T13, T18, and T21, twice as many of these aneuploidies are picked up using NIPT, compared with the liveborn prevalence. This discordance is even more pronounced for sex chromosome aneuploidy (SCA), where more than 90 times more abnormal test results are found than abnormally born children are identified. NIPT has been suggested to have a role in the evaluation for etiology in first- and second-trimester fetal loss [17,18]. Expanded testing can provide an explanation for miscarriage or unexplained loss. Expanded NIPT is a valuable option for couples where one of the partners carries a balanced reciprocal or Robertsonian translocation. PPV is lowest when the NIPT results are compared with the fetal or newborn genotype and highest when the NIPT test results are compared with the placental genotype.

### 3.5. Test Statistics (False-Positive Rate)

Non-invasive prenatal testing has a much lower false-positive rate than prior methods of prenatal screening, with the rate for targeted screening panels being approximately 0.13% when considering CAT. Expansions of screening panels to include sex chromosome aneuploidy, rare autosomal trisomy, or microdeletion/microduplication syndromes (MMSs) to analyze the entire fetal genome (as opposed to exclusively targeting chromosomes 21, 18, or 13) have resulted in an increase in the number of women obtaining high-risk results despite carrying a euploid fetus, prompting the U.S. Food and Drug Administration to issue a statement of caution regarding the interpretation of high-risk screening results [19,20].

The fetal fraction is the ratio of cell-free fetal DNA to total cell-free DNA, and the minimum threshold for fetal fraction to yield an informative result is typically around 4% [17,21]. However, the calculation methods for fetal fraction can differ among laboratories, and some labs may not accurately calculate the fetal fraction [17]. In a male fetus, the fetal fraction can be directly imputed from the percentage of reads mapping to the Y chromosome. In a female fetus, there are several techniques and algorithmic approaches to the calculation of the fetal fraction. This includes both unique polymorphisms or short tandem repeats that differ between the mother and the fetus, as well as morphologic discrimination between the fragment sizes.

Because “fetal” (placental) cfDNA is generally shorter than maternal cfDNA, maternal plasma samples with a higher fetal DNA concentration would have a higher proportion of short DNA fragments. The size distribution histogram of maternal origin cell-free DNA fragments shows a major spike at 166 base pairs (bps), corresponding to the length of DNA wrapped around a single nucleosome core (∼147 bp) and the adjacent linker regions of ∼20 bp) [22]. The cell-free DNA size distribution of placental origin shows a reduced 166 bp peak and a major peak is observed at 143 bp corresponding to the length of DNA around a nucleosome core only. This difference can be used to differentiate relative contributions in the maternal sample and enhance the fetal fraction before sequencing. On the other end of the spectrum, high fetal fraction (around 35.3%) may be an indicator of obstetric conditions like an abnormally invasive placenta and significantly increased fetal fraction may be associated with spontaneous preterm birth.

### 3.6. Non-Reportable Results

A small percentage of tests will not be informative. The test failures occur for a variety of reasons related to technical aspects of the assay that prevent a clear delineation between likely normal and likely aneuploid. Assay quality control criteria, including cfDNA extraction, library construction, sequencing, and fetal DNA concentration (<4%), are all reasons that a test may fail to yield informative results. Samples that did not meet the quality control criteria for GC content (outside 38–42%), insufficient unique reads mapping to the hg19 human reference genome (below 3.5 million), or fetal DNA concentration are usually reported as a “no call.” An insufficient amount of total or placental DNA can affect the algorithm’s predictive accuracy. If the gestational age at the time of the sample collection of the NIPT is too early the fetal fraction will be too low to report a reliable risk assessment [23]. Insufficient fetal fraction is found in several other situations, including elevated maternal BMI, increased maternal age, and autoimmune conditions, such as antiphospholipid syndrome [24,25]. Heparin administration is linked to lower fetal fraction, ultimately affecting the NIPT results. Heparin is thought to increase the maternal cfDNA as well as decrease placental apoptosis, which can affect NIPT analysis [26].

Although the NGS test has high sensitivity and specificity, false-positive and false-negative results do occur and have various causes, including maternal and placenta conditions such as mosaicism, maternal copy number variation (CNV), maternal malignancy, uterine fibroids, and vanishing twin syndrome [27]. Fetal fraction and confined placental mosaicism are the most frequently encountered reasons for the test to either fail to yield informative results or to report results that are discordant with the fetus (Table 1). Gil et al. specifically described the NIPT failure rates in a meta-analysis, demonstrating a combined false-positive rate of 0.13% for trisomies 21, 18, and 13 [28].

Vanishing twin syndrome can cause NIPT to detect the aneuploidy that resulted from the deceased co-twin, leading to a discordance between the genotype detected on the screening test and the phenotype in the remaining, living fetus. Maternal organ/tissue transfer from a male donor can cause errors in fetal sex determination.

Maternal conditions that can cause discordant results include uterine leiomyomas, which are present in about 11% of pregnant patients. Large fibroids are often aneuploid, and can release abnormal cfDNA fragments, resulting in a false-positive NIPT [24]. Women with fibroids were nearly twice as likely to have a false-positive result for MMS, and this was associated positively with both fibroid number and volume. The presence of fibroids did not appear to influence the accuracy of genome-wide NIPT for the common autosomal trisomy or sex abnormalities [29]. The most common genetic anomaly in fibroids is del 7q22q32, accounting for 17% of karyotypically abnormal fibroids (Table 1).

Mosaicism, from errors in meiosis or mitosis, can be seen in just the fetus, just the placenta, or both the placenta and fetus, known as feto-placental mosaicism [27,30,31]. Mosaicism can lead to both false-positive and false-negative results depending on the status of the fetus and placenta. Increased experience with these conditions has spurred improvements in the data analytic tools, which in turn improves the accuracy of the NIPT results for predicting the fetal condition. The Reanalysis of the Comparison of Aneuploidy Risk Evaluations (CARE) study, using an updated analytic algorithm applied in the Illumina clinical laboratory since 2013, was able to correctly reclassify technical and biological reasons for the original false-positive results and the reanalysis of the sample with the improved algorithm reduced the FPR by reclassifying outlier results, including fetoplacental mosaicism and variants in maternal copy number. Continuous improvements in informatics improved the test performance in cfDNA sequencing [32].

### 3.7. Confined Placental Mosaicism

Chromosomal abnormalities confined to the placenta, with a normal fetal chromosomal profile, are known as confined placental mosaicism (CPM) and can be the cause of some false-positive NIPT results [33]. CPM has been demonstrated to affect about 1–2% of pregnancies [24,34]. CPM is one of the most widely recognized causes of false-positive noninvasive prenatal testing (NIPT) results. Cell-free ‘fetal’ DNA in maternal blood originates from the cytotrophoblast and does not always match true fetal DNA. Therefore, achieving 100% sensitivity and specificity for NIPT is not possible, and both positive and negative results must be interpreted in light of all the available clinical data.

Although about half of pregnancies with CPM will be uneventful, pregnancies with CPM are at increased risk for complications such as fetal growth restriction, low birth weight, and maternal hypertensive disorders. Pregnancy outcomes in cases of CPM are dependent on the origin of the aneuploid cells, with meiotic trisomy being significantly associated with adverse pregnancy outcomes. Similarly, pregnancies with a low PAPP-A or high levels of free β-hCG have a higher rate of adverse pregnancy outcomes in cases of CPM. Depending on how the CPM was ascertained and which chromosome was affected, the risk of an abnormal pregnancy outcome may be high. In one study, FGR was reported in 71.7% of CPM cases, and preterm birth (<37 weeks of delivery) was reported in 31.0% of cases. A high rate of structural fetal anomalies, 24.2%, in cases with CPM was also identified. High levels of mosaicism in CVS and the presence of uniparental disomy (UPD) were significantly associated with adverse pregnancy outcomes. When confined placental mosaicism occurs, both NIPT and invasive diagnostic methods may detect chromosome abnormalities that are not reflected in the fetus.

CPM can be divided into three subtypes (types 1, 2, and 3) based on the affected trophoblastic cell. CPM type 1 is referred to when the chromosome abnormality is only found in the cytotrophoblast (CTB), whereas type 2 is restricted to an abnormality in the mesenchymal core (MC) of the chorionic villi. If an abnormal karyotype is present in both cell layers, MC and CTB, it is called type 3. Since the cytotrophoblast is the predominant cell contributing to the “fetal” fraction, only types 1 and 3 are usually detected through NIPT. Type 2 is found in CVS. If CPM involves a trisomy, this chromosome abnormality may originate from either meiosis or mitosis. Some suggest that an association exists between adverse pregnancy outcomes and CPM for trisomy 16. Trisomy 7 on NIPT is almost always CPM. A mosaic level under 30% is likely to be missed by NIPT. However, this largely depends on fetal fraction, with 100% of trisomy potentially going undetected if the fetal fraction is too low, and a mosaic of ≤ 30% being detected if the fetal fraction is adequately high.

Aneuploidy seemingly confined to the placenta (CPM) is not harmless. Published data on expanded NIPT are available on more than 450,000 pregnancies. In almost 60% of RAT (90/153), either the aneuploidy was confirmed, the fetus had an abnormal phenotype, or there was an adverse pregnancy outcome [33,35,36]. When confined placental mosaicism occurs, both NIPT and invasive diagnostic methods may fail to detect chromosome abnormalities. In this case, increasing the sequencing depth does not improve the detection rate.

### 3.8. Mosaic Ratio

Rafalko and colleagues described the novel metric of the mosaicism ratio (MR). The mosaic ratio is the proportion of placental cfDNA that is aneuploid in maternal blood. Lower mosaicism ratios were more likely to exhibit placental mosaicism or other sources of false-positive results, such as vanishing twin syndrome [31]. Since CPM involving meiotic trisomy is more likely to have high levels of mosaicism in the cytotrophoblast (CTB), it will lead to an increased amount of trisomic cfDNA in maternal blood. A higher mosaic ratio is therefore expected when there is an association between the mosaic ratio and adverse pregnancy outcomes in cases of CPM. CPM involving a meiotic trisomy has a significantly higher mosaic ratio than CPM involving a mitotic trisomy.

As previously discussed, there is an increased risk of aneuploidy with a no-call on NIPT. To avoid delay in diagnosis, invasive testing should be offered if a test fails to yield informative results. When the patient elects instead to repeat the test, most no calls will eventually yield informative results about 75–80% of the time [37]. Nearly 2% of samples will show a fetal fraction <4% and in these cases, the rates of T18 and T13, but not T21, are higher than in those pregnancies with a successful test. Therefore, patients with failed tests owing to low fetal fraction at the second redraw have a higher a priori risk for T18 and T13 and, as such, necessitate specific healthcare management, including a detailed sonographic investigation, a non-CF DNA-based screening test (gestational age permitting), or an invasive procedure.

Because most cfDNA originates from the mother, copy-number variants in the maternal genome (mCNVs) can cause sufficiently large depth deviations to yield false positives, thereby reducing the specificity of NIPT (Table 1). While newer algorithms have reduced false positives, they have not eliminated them altogether [32]. mCNVs are common on the CAT chromosomes that NIPT interrogates (4.5% of patients have mCNV on chromosome 13, 18, or 21), and these can cause false positives if not properly filtered at the algorithmic level [38]. Two recent studies of trisomy 13, 18, and 21 attributed one-third to one-half of NIPT false positives to maternal duplications. A 22-study meta-analysis of NIPT discordances found that 48% of false positives with an identified cause were due to mCNVs.

If there is a question about the origin of the NIPT abnormality, repeating the NIPT after delivery to see if the abnormality is still present can help to determine if the abnormality was of maternal origin [33]. A rare biological cause of false-positive NIPT results is maternal neoplasia, which may have chromosomal abnormalities and release cfDNA into the maternal blood.

CF DNA is not diagnostic and should not replace invasive testing when there is a high suspicion of an abnormality, such as when a structural abnormality is detected on an ultrasound. Patients should not assume that a ‘low-risk’ NIPT alone is sufficient evaluation in the presence of an ultrasound anomaly. An invasive diagnostic procedure should be offered after any positive screen or test failure due to the higher prevalence of aneuploidy. Furthermore, a negative NIPT result with the presence of sonographic abnormalities always needs to be checked using an invasive diagnostic procedure.

### 3.9. Late First-Trimester Ultrasound

Late first-trimester ultrasound (LFTU) findings significantly affected the PPV of the NIPT result for trisomy 13 (T13), 18 (T18), 21 (T21), monosomy X (MX), and RAT but not for the other sex chromosomal abnormalities or segmental imbalances (>7 Mb). Structural abnormalities on LFTU and/or increased NT (>2.2 mm at 10–11 weeks) require genetic counseling to discuss if NIPT should be bypassed in favor of CVS.

Abnormal LFTU increased the PPV of the NIPT screen close to 100% for T13, T18, T21, MX, and RAT. When the LFTU was normal, the incidence of confined placental mosaicism (CPM) was highest in pregnant women with a high-risk NIPT result for T13, followed by T18 and T21. After normal LFTU, the PPV for T21, T18, T13, and MX decreased to 68%, 57%, 5%, and 25%, respectively [39]. Genome-wide NIPT (gwNIPT) using massive parallel shotgun sequencing enables the detection of chromosomal anomalies beyond the common autosomal trisomies (CAT) (T13, T18, and T21). Rare autosomal trisomies (RAT) are generally incompatible with life in their non-mosaic form, but fetal mosaicism or uniparental disomy (UPD) can result in ongoing pregnancies with a range of fetal structural, growth, and neurodevelopmental abnormalities [36]. Most high-risk NIPT–RAT results are false positives, but the information can still be useful in clinical management, as the aneuploid placenta—which may lead to a false-positive result—can subsequently cause fetal growth restriction. The positive predictive value of cell-free DNA in diagnosing rare autosomal trisomy is approximately 11% when predicting an affected fetus. It appears that the more severe a genetic condition, the larger the decrease in PPV when LFTU is normal. High-risk NIPT results for the more severe genetic abnormalities with a sonographically normal fetus were associated with a higher rate of CPM, particularly a high-risk NIPT result for T13 [40]. When monosomy X resulted on NIPT despite a normal LFTU, most fetuses were either 46XX or had mosaicism (3–63% mosaic). Conversely, abnormal ultrasound findings showed a true-positive non-mosaic MX result in 90% of cases [39]. The high PPVs of NIPT for T21 (87%) and T18 (80%) are not sufficiently modified by normal LFTU findings (68% and 57%, respectively) to alter management. Abnormal LFTU has a powerful impact on the PPV of NIPT for MX and RAT.

### 3.10. Cancer Detection

Liquid biopsy is a tool that uses the NGS platform to investigate for the presence of circulating tumor cfDNA as a minimally invasive diagnostic method to identify malignancy, since up to 90% of solid tumors and more than half of blood tumors are aneuploid [41] (Table 1). Giudice and colleagues demonstrated cfDNA tumor DNA in non-pregnant patients with known cancer, either at diagnosis or relapse, and 50% displayed abnormalities on NIPT [42]. Schrag and colleagues reported the results of the PATHFINDER study, a multicancer early detection (MCED) blood test that can identify a cancer signal from circulating cfDNA [43]. This prospective cohort study investigated the feasibility of MCED testing for cancer screening, evaluating methylation patterns as a marker for cancer. Experience has demonstrated that some patterns of unusual results on prenatal NIPT also suggest the increased risk of maternal malignancy. When whole-genome sequencing reveals unusual findings, such as autosomal monosomy, multiple aneuploidies, or chaotic patterns of gain and loss across multiple chromosomes, there is strong clinical suspicion for malignancy.

The most common cancers to occur during pregnancy are typically Hodgkin’s and non-Hodgkin’s lymphoma, leukemia, breast, ovarian, cervical, melanoma, and colon cancers [6,10]. Certain cytogenetic patterns have been associated with specific malignancies. Chromosomal MMS gains of 2p, 5p, and 9p and losses of 11q, 6q, and 7q have been identified in classical Hodgkin’s lymphoma [44]. Population-based screening in the Netherlands and Belgium was reported in the TRIDENT-2 study. Among the 231,896 pregnant women in the study, 48 had a genome-wide NIPT result assessed as indicative of a maternal malignancy. Of those 48 patients, 23 (48%) had multiple chromosomal aberrations detected. For the 23 patients with multiple aberrations, a confirmed malignancy was diagnosed in 16 cases. This corresponds to a confirmed malignancy rate of nearly 70% (16/23) This study recommended further investigation, including hematologic blood tests and total-body MRI, in order to confirm maternal malignancy during pregnancy [45]. The Maastricht criteria, which consider various factors, including the number of chromosomal aberrations, monosomy, certain trisomy, and the total number of trisomy, assign a score based on each criterion. The cumulative score correlates to the likelihood of an underlying malignancy. This presumes that the fetus and placenta have been evaluated after invasive testing, such as amniocentesis or chorionic villus sampling, and advanced ultrasound has ruled out fetal disease [44]. The Incidental Detection of Maternal Neoplasia Through Non-Invasive Cell-Free DNA Analysis (IDENTIFY) trial in asymptomatic pregnant women who underwent NIPT showed an incidence of cancer in 48.6% (52/107) of participants referred for abnormal NIPT results that were suspicious for maternal malignancy. Of those 52 participants, 49 had multiple copy number gains and losses across various chromosomes. The remaining 55 patients had no identifiable cause for their abnormal results, raising questions about potential long-term cancer risk [11]. It is hoped that the follow-up from this study will aid in developing a consensus on how to manage asymptomatic patients who exhibit suspicious results on cfDNA testing, suggesting an increased risk of maternal cancer.

Cell-free DNA technology continues to make advances in screening and diagnosis for numerous conditions. Laboratories that provide such testing compete for market share by emphasizing the performance of their platforms in new or evolving aspects, such as rare autosomal trisomies, microdeletions, and pathogenic copy number variant detection, or by refining processing methodologies to enhance fetal fraction. Practitioners who order such testing should be familiar with their preferred laboratory’s platform methodology, its test performance, the myriad biological explanations for phenotype–genotype discordance, and the reporting strategy for unexpected results. When such unexpected NIPT results occur, it is also incumbent on providers to have an established patient counseling strategy, as such results may indicate fetal, placental, or maternal abnormalities.

There is strong clinical suspicion for malignancy when whole-genome sequencing reveals unusual findings, such as autosomal monosomy, multiple aneuploidies, or chaotic patterns of gain and loss across multiple chromosomes. The Incidental Detection of Maternal Neoplasia Through Non-Invasive Cell-Free DNA Analysis (IDENTIFY) trial in asymptomatic pregnant women who underwent NIPT showed an incidence of cancer in 48.6% (52/107) of participants referred for abnormal NIPT results that were suspicious for maternal malignancy. Of those 52 participants, 49 had multiple copy number gains and losses across various chromosomes. The remaining 55 patients had no identifiable cause for their abnormal results, raising questions about potential long-term cancer risk [11]. It is hoped that the follow-up from this study will aid in developing a consensus on how to manage asymptomatic patients who exhibit warning signs on cfDNA testing that are suggestive of an increased risk of maternal cancer. As more information unfolds, it will be important to assess the predictive value of this testing for cancer, considering that the estimated risk of cancer during pregnancy is only 1 per 1000 patients.

## 4. Conclusions

Cell-free DNA technology continues to evolve, and practitioners must be aware of factors that can affect test results. Diagnostic testing is recommended when there is a positive NIPT result, a no-call or test failure, and when there is an abnormality on an ultrasound, even with a “low-risk NIPT” result. As our case illustrates, cfDNA may offer useful information about the presymptomatic recurrence of a previously diagnosed cancer in a woman who has subsequently become pregnant.

## Figures and Tables

**Table 1:** Causes of NIPT discordant results.

Category	Mechanism/Cause	NIPT Pattern
Insufficient Fetal Fraction	Elevated maternal BMI, increased maternal age, autoimmune conditions, heparin administration	No call, inconclusive, failed, incomplete
Technical Error	Do not meet quality control criteria, low fetal fraction (less than 4%), early gestational age	No call, inconclusive, failed, incomplete
Vanishing Twin Syndrome	NIPT detects aneuploidy of the deceased co-twin	Single aneuploidy
Maternal Copy Number Variation	Copy number variations in the maternal genome	Deletions and duplications
Confined Placental Mosaicism	Aneuploid cytotrophoblast (CTB), mesenchymal core (MC), or both	Specific chromosomal aneuploidy
Uterine Leiomyomas	Fibromas release abnormal cfDNA fragments	No call or genetic anomaly, most commonly del 7q22q32
Maternal Malignancy	NIPT detects circulating tumor cfDNA	Multiple aneuploidies

## Data Availability

The original contributions presented in this study are included in the article. Further inquiries can be directed to the corresponding author.

## Abbreviations

The following abbreviations are used in this manuscript:

NIPT	Non-invasive prenatal testing
cfDNA	Cell-free DNA
NG	Next-generation sequencing
PPV	Positive predictive value
NPV	Negative predictive value
CAT	Common autosomal trisomy
RPS	Reads per sample
SNP	Single nucleotide polymorphism
RCA	Rolling circle amplification
RAT	Rare autosomal trisomy
SCA	Sex chromosome aneuploidy
MMS	Microdeletion/microduplication syndrome
CNV	Copy number variation
CPM	Confined placental mosaicism
CTB	Cytotrophoblast
MC	Mesenchymal core
LFTU	Late first-trimester ultrasound
gwNIPT	Genome-wide NIPT
UPD	Uniparental disomy
MCED	Multicancer early detection
ICE	Ifosfamide, carboplatin, and etoposide
BEAM	Carmustine, etoposide, cytarabine, and melphalan
